# Phytochemistry, Antioxidant Activity, Antiproliferative Effect, and Acute Toxicity Testing of Two Moroccan *Aristolochia* Species

**DOI:** 10.1155/2019/9710876

**Published:** 2019-12-03

**Authors:** Mohammed Bourhia, Fatima Ezzahra Laasri, Samir Iben Moussa, Riaz Ullah, Ahmed Bari, Syed Saeed Ali, Aghmih Kaoutar, Amal Ait Haj Said, Mohammed El Mzibri, Gmouh Said, Naima Khlil, Laila Benbacer

**Affiliations:** ^1^Laboratory of Chemistry, Biochemistry, Nutrition, and Environment, Faculty of Medicine and Pharmacy, University Hassan II, Casablanca, Morocco; ^2^Research Unit and Medical Biology, National Center for Nuclear Energy, Science and Technology (CNESTEN), Rabat 10001, Morocco; ^3^Laboratory of Nutrition, Health and Environment, Faculty of Sciences, Ibn Tofail University, Kenitra, Morocco; ^4^Medicinal Aromatic and Poisonous Plants Research Center, College of Pharmacy, King Saud University, P. O. Box 2457, Riyadh 11451, Saudi Arabia; ^5^Central Laboratory, College of Phamacy, King Saud University, P. O. Box 2457, Riyadh 11451, Saudi Arabia; ^6^Laboratory REMTEX, Higher School of Textile and Clothing Industries, Casablanca, Morocco; ^7^Laboratory GeMEV, Faculty of Sciences Aïn Chock, Hassan II University, Casablanca, Morocco; ^8^Laboratory of Pharmacognosy, Faculty of Medicine and Pharmacy of Casablanca, Hassan II University, Casablanca, Morocco; ^9^Laboratory of Engineering and Materials LIMAT, Faculty of Sciences Ben M'Sik, University Hassan II, Casablanca, B. P. 7955, Morocco

## Abstract

**Ethnopharmacological Relevance:**

*Aristolochia baetica* (*A. baetica*) and *Aristolochia paucinervis* (*A. paucinervis*) have been largely used in Moroccan folk medicine. The objective of the study was to investigate the phytochemical composition, the antioxidant activity, the antiproliferative effect, and the acute toxicity of the methanolic extract of *A. baetica* and *A. paucinervis* roots.

**Materials and Methods:**

Phytochemical composition of the methanolic extract of *A. baetica* and *A. paucinervis* roots were studied using qualitative and quantitative methods, the antioxidant activity was evaluated using DPPH assay, the antiproliferative effects against human cancer cell lines (T-24, HT-29, and Hep G-2) was assessed using WST1 assay, and the acute toxicity was carried out orally by gavage of single dose 2000 mg/kg to mice for 14 days.

**Results:**

The two studied plants have different classes of secondary metabolites. The concentrations of the total polyphenolic content of *A. baetica* and *A. paucinervis* root extracts were estimated at 360 ± 20 mg GAE/g and 280 ± 27 mg GAE/g, respectively. The total flavonoids content of *A. baetica* and *A. paucinervis* extracts were estimated at 35 ± 8 mg QE/g and 235 ± 7 mg QE/g, respectively. *A. baetica* and *A. paucinervis* extracts exhibited promising DPPH activity with IC_50_ values of 150 ± 8 *μ*g/ml and 160 ± 10 *μ*g/ml, respectively. The extracts exerted also antiproliferative effects on all tested cancer cell lines (T-24, HT-29, and Hep G-2) with IC_50_ values ranging from 6 ± 1 *μ*g/ml to 380 ± 7 *μ*g/ml. Regarding the results of acute toxicity study, no signs of toxicities nor mortalities were observed on the oral treated mice with 2000 mg/kg of the two investigated exacts.

**Conclusion:**

The methanolic extracts of *A. baetica* and *A. paucinervis* possess several phytochemicals that exhibited promising free radical scavenging activity and antiproliferative effects.

## 1. Introduction

Sine antiquities, plants have played an important role in our daily lives as they have been a source of food, fiber, and other necessary needs. Among these resources, medicinal plants have been widely used to protect humans against serious diseases [1, 2]. They are considered as a natural reservoir of biologically active substances with different biological properties. These bioactive natural remedies are increasingly used to treat a wide variety of clinical diseases because of its lower side effects [3].

Currently, therapeutic chemistry has great interests in health care, with almost 80% of the population throughout the world using medicinal plants [4]. As a result, several research studies have paid more attention to medicinal plants as an important source of chemical compounds and bioactive substances with antibacterial, anti-inflammatory, antioxidant, and anticancer activity [5]. Some of the natural substances have been recognized as key players in pharmacology for the development of new drugs used in the treatment of cancer [6]. Scientific research on plants used in traditional medicine is widely required to ensure safety control and to avoid potential risks related to the ingestion of toxic herbs. Data collected from *in vitro* and *in vivo* toxicological studies of herbs are needed to consolidate the scientific validity of herbs [7].

*Aristolochia* species have been widely used in Moroccan traditional medicine to treat various diseases for many years ago [7]. Nowadays, some of these plants are used to treat cancer [8], digestive diseases [9], rheumatic, abortifacient, and cutaneous neoplasm. They are also used as a stimulant of blood circulatory as anti-inflammatory and antiseptic [10].

The present work was conducted to determine the scientific basis of traditional uses of *A. baetica* and *A. paucinervis*. It aims at the investigation of the phytochemical composition, the antioxidant activity, the antiproliferative effect, and the acute toxicity of the methanolic extract of the two reported plants.

## 2. Materials and Methods

### 2.1. Plant Material

*A. baetica* was collected in January 2016 from the surrounding region of Chefchaouen (Moroccan city). *A. paucinervis* was harvested in December 2016 at 30 km East of Khouribga (Moroccan city). The collected plants were identified by Dr. Mohammed Fanane (Department of Botany, Scientific Institute of Rabat, Morocco). A voucher specimen has been deposited in the herbarium under 1045 and 1046, respectively. The roots of both, *A. baetica and A. paucinervis* were initially cleaned, washed with water, dried in the shade, and chopped into small pieces using an electric mixer.

### 2.2. Preparation of Plant Extract

25 g of dried powder of *A. baetica* and *A. paucinervis* roots was extracted using Soxhlet at 40°C for 2 h using methanol as an extraction solvent. The obtained extract was centrifuged, filtered, and evaporated at low pressure (40°C) to remove the excess solvent in order to obtain 3 g of crude extract.

### 2.3. Phytochemical Screening

The plant materials were subjected to qualitative phytochemical careening in order to qualitatively determine some type of interesting constituents that could be responsible for biological activities. Alkaloids, flavonoids, polyphenols, anthraquinones, saponins, tannins, sterols, and terpenes were the major checked groups using standard methods as reported in earlier literature with slight modifications [11].

### 2.4. Identification of Bioactive Constituents by GC-MS

GC-MS analysis of the methanolic extracts of the studied plants were performed using a Claus 580 Gas Chromatography according to the following acquisition parameters: oven: initial temp 50°C for 2 min, ramp 11°C/min to 200°C, hold 0 min, ramp 6°C/min to 240°C, hold 1 min, split = 10 : 1, carrier gas = He, solvent delay = 4.00 min, transfer temp = 280°C, source temp = 250°C, scan: 40 to 450 Da, and column 30.0 m × 250 *μ*m.

### 2.5. Total Polyphenolic Content

The total phenolic content of *A. baetica* and *A. paucinervis* extracts was determined using the Folin–Ciocalteu method and gallic acid as standard. Aliquots of test samples (250 *μ*L) of a diluted solution of extracts were mixed with 1.5 mL of sodium carbonate solution (7.5%). After 5 min, 1.25 mL of Folin–Ciocalteu reagent, reveal the composition of this reagent (0.2 N), was added to the mixture, allowed to stand at room temperature for 30 min in darkness. The reading was carried out versus a blank at 765 nm. The calibration curve was plotted using gallic acid as a positive control. The results were expressed as gallic acid equivalent per gram of dry extract (mg GA E/g) [12].

### 2.6. Total Flavonoid Content

The total flavonoid content of *A. baetica* and *A. paucinervis* extracts was identified using the aluminum chloride assay (colorimetry). Each diluted sample was mixed with 1.5 mL of AlCl_3_ (2%) and incubated for 60 min at room temperature. The absorbance was read spectrophotometrically against a blank at 415 nm. Quercetin was used as a reference standard compound. The calibration curve was plotted using quercetin as a standard compound. The results were expressed as quercetin equivalent per gram of dry extract (mg QE/g) [13].

### 2.7. Antioxidant Activity

The antioxidant activity of *A. baetica* and *A. paucinervis* extracts was evaluated *in vitro* using DPPH (2,2-diphenyl-1-picrylhydrazyl) assay according to the method described in previous literature [14]. About 1 ml of methanolic extract at different concentrations ranging from 100 *μ*g/ml to 900 *μ*g/ml was mixed with 500 *μ*l of methanolic solution of DPPH (0.005%). After 30 min of incubation in darkness at room temperature, the absorbance (*A*) of the control and samples was read spectrophotometrically at 517 nm. Ascorbic acid was used as a positive control. The free radical scavenging activity was calculated in percentage as follows:

(1) $$\begin{array}{c} \text{Free radical scavenging activity}\left(\%\right)=\frac{\left(A\right)\text{control}–\left(A\right)\text{sample}}{\left(A\right)\text{control}}*100. \end{array}$$

IC_50_ was determined graphically from the graph plotting inhibition percentage against extract [15].

### 2.8. *In Vitro* Cytotoxicity Assay

#### 2.8.1. Cell Culture and Treatment

Three Cancerous cell lines were selected to be tested including human bladder cancer cell lines (T-24). The latter were cultured in McCoys5a; in parallel, the liver cancer cell lines (Hep G-2) and human colon cancer cell lines (HT-29) were cultured in DMEM media added with 10% heat-inactivated fetal calf serum, antibiotics (1%), and glutamine (1%). The cancerous cells were grown at 37°C in a humidified incubator set at 5% CO_2_. The cultures were left until they formed a monolayer on the flask. The cells were washed with PBS and trypsinized in order to be detached and then adding a complete medium to inhibit the reaction.

Antiproliferative activity of the methanolic extract of *A. baetica* and *A. paucinervis* against T-24, Hep G-2, and HT-29 cell lines was assessed on the basis of mitochondrial metabolic activity using WST1 (disodium mono{4-[3-(4-iodophenyl)-2-(4-nitrophenyl)-2H-tetrazol]-3-ium-5-yl]benzene-1,3 disulfonate}). During the exponential growth of the tested cells in 96-well microplates at a density of 8000 cells per well, the seeding medium was removed and replaced by extracts prepared in medium to concentration up (0 to 400 *μ*g/mL). WST1 assay was performed in triplicate, and the mitomycin was used parallelly as positive control. After 72 h of incubation, briefly 100 *μ*l of the medium was removed from each well and 10 *μ*l of WST1 solution was added to cultured cells. The plate was incubated again for 4 h at 37°C in a dark wet atmosphere. The absorbance was read at 590 nm using a Wallac Victor X3 multiwell spectrophotometer.

The percentage of cytotoxicity was defined according to the following formula:

(2) $$\begin{array}{c} \text{cell death}\left(\%\right)=\frac{\text{control OD}-\text{sample OD}}{\text{control OD}}\;*\;100. \end{array}$$

The percentage of viability was calculated according to the following equation:

(3) $$\begin{array}{c} \text{cell viability }\left(\%\right)=100-\left(\%\text{ cell death}\right). \end{array}$$

IC_50_ value (the inhibition concentration required to reduce 50% of cell proliferation) was determined graphically on the basis of regression analysis performed on WST1 assay viability [16].

### 2.9. Animal Material

Adult Swiss albino mice weighing approximately 25 g were used for acute toxicity. The mice were purchased from the animal colony of Pasteur Institute (Casablanca, Morocco). All animals were kept in polypropylene cages. The animals were acclimatized for one week under laboratory conditions of regular light/dark cycles (12/12 h) and temperature (24 ± 2°C). The animals had free access to tap water and a normal pellet diet [17].

### 2.10. Study of Acute Toxicity

Nine male adult mice were randomly divided into three experimental groups of three mice each. The animals were grouped in polypropylene cages and fasted for 12 h. The organic extract of the studied plants was administered to each treatment group once as follows:

  Control group—treated with vehicle (distilled water)
  Group A—*A. baetica* extract (2000 mg/kg)
  Group B—*A. paucinervis* extract (2000 mg/kg)

Animals were observed for signs of toxicity, mortalities, changes in general behavior, and physical appearance. This study was conducted according to the Organization for Economic Cooperation and Development (OECD) Guidelines No. 425 [18].

### 2.11. Statistical Analysis

Data for each test are the average of triplicate experiments ± standard deviation (SD), and statistical analysis of difference was performed using ANOVA. The means were compared using the Holm–Sidak Test. Statistically *p* value less than 0.05 was considered to indicate significance.

## 3. Results

### 3.1. Phytochemical Screening

The phytochemical screening of *A. baetica* and *A. paucinervis* roots revealed the presence of flavonoids, polyphenols, alkaloids, tannins, saponins, and the absence of anthraquinone, sterols, and terpenes (Table 1).

### 3.2. GC-MS Analysis

**Figure 1:**
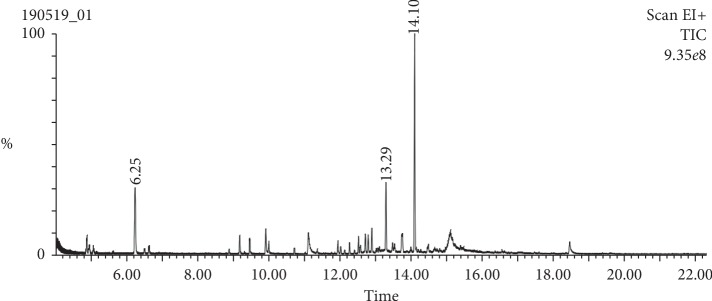
GC-MS spectral chromatogram of *A. baetica* methanolic extract.

The GC-MS analysis of *A. baetica* methanolic extract revealed ten compounds: pseudocumene, tetracyclo [3.3.1.0.1(3,9)]decan-10-one, *p*-vinylguaiacol, 2-epi-trans-*β*-caryophyllene, guaia-6,9-diene, pacifigorgiol, maaliol, methylglucose, isoaromadendrene epoxide, and *trans*-sinapyl alcohol (Figure 1; Table 2).

**Figure 2:**
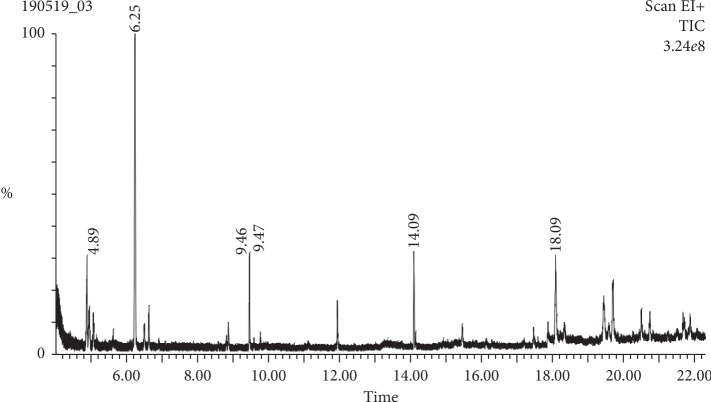
GC-MS spectral chromatogram of *A. paucinervis* methanolic extract.

Regarding *A. paucinervis* methanolic extract, the GC-MS identified seven compounds: dihydro-4,4,5,5-tetramethyl 2(3H)-furanone, mesitylene, dodecane, maaliol, 2-palmitoylglycerol, dioctyl terephthalate, phthalic acid, and di(2 propylpentyl) ester (Figure 2; Table 3).

### 3.3. Total Polyphenolic and Flavonoid Contents

Calculations of total polyphenolic content in the organic extract of the studied plants were based on the equation obtained from the standard gallic acid graph. Total polyphenolic contents of 360 ± 20 mg GAE/g and 280 ± 27 mg GAE/g were found in *A. baetica* and *A. paucinervis* methanolic extracts, respectively. Regarding the total flavonoid content calculation, the formula obtained from the standard quercetin graph was applied. Total flavonoid content of 35 ± 8 mg QE/g was obtained with *A. baetica* extract and 235 ± 7 mg QE/g with *A. paucinervis* extract.

### 3.4. Antioxidant Activity

**Figure 3:**
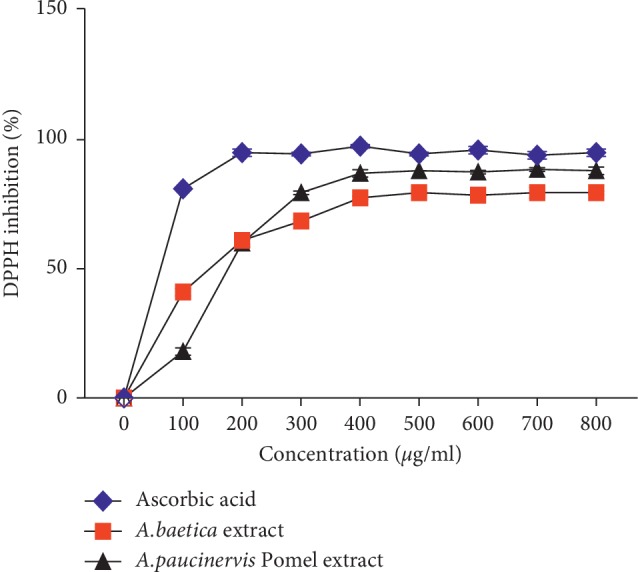
DPPH radical scavenging activity of methanolic extract of *A. baetica* and *A. paucinervis*.

The antioxidant activity of *A. baetica* and *A. paucinervis* methanolic extracts was evaluated by the DPPH free radical scavenging test. As shown in Figure 3, both extracts exhibit promising antioxidant activity in a concentration-dependent manner.

The IC_50_ value (the inhibitory concentration of extract required to inhibit 50% of the initial DPPH free radical) of the plant extracts and ascorbic acid as a positive control was determined graphically from the graph of DPPH inhibition percentage. The results of IC_50_ values of methanolic extract of *A. baetica*, *A. paucinervis*, and ascorbic acid were determined approximately at 150 ± 8 *μ*g/ml, 160 ± 10 *μ*g/ml, and 25 ± 3 *μ*g/ml, respectively. In order to perform the comparison, the IC_50_ values of DPPH radical scavenging activity of methanolic extract of *A. baetica* and *A. paucinervis* showed a significant difference compared to DPPH IC_50_ value of ascorbic acid as a standard (*p*^*∗∗∗*^ < 0.05).

### 3.5. *In Vitro* Cytotoxicity Assay

**Figure 4:**
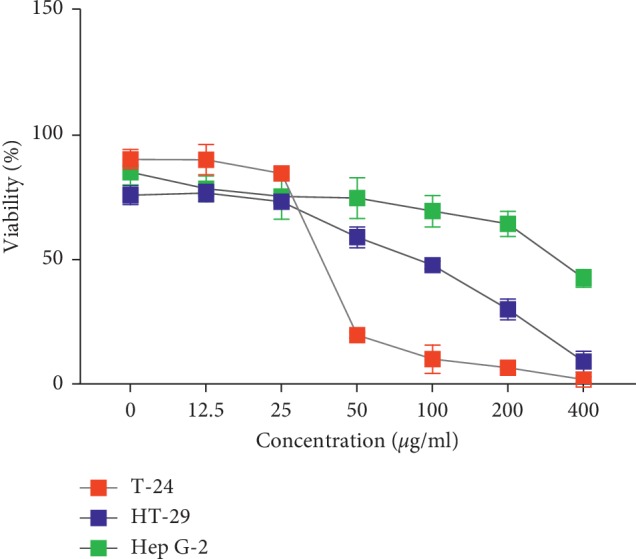
Cell viability after 72 h of treatment with the methanolic extract of *A. baetica* roots.

**Figure 5:**
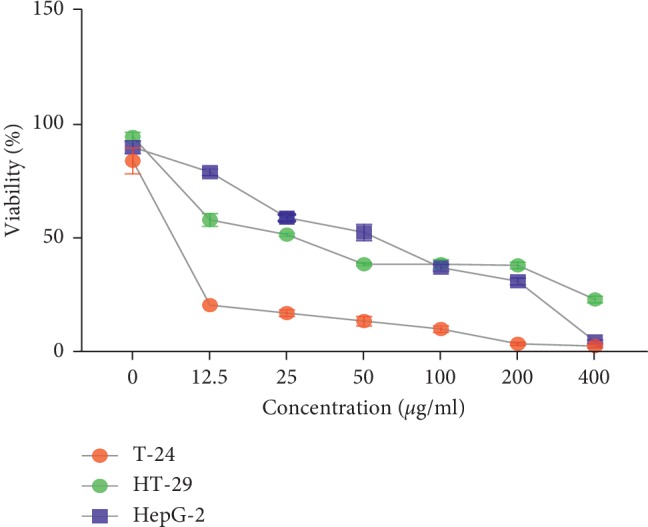
Cell viability after 72 h of treatment with the methanolic extract of *A. paucinervis* roots.

The methanolic extract of *A. baetica* and *A. paucinervis* was investigated for potential antiproliferative effects on T-24, Hep G-2 and HT-29 human cancerous cell lines using WST1 assay. Figures 4 and 5 show important antiproliferative effects induced by the investigated extracts on the treated cells in a time- and concentration-dependent manner.

Regarding data presented in Figure 4, the methanolic extract of *A. baetica* roots induced antiproliferative effects on T-24 and HT-29 cell lines with IC_50_ 48 ± 5 *μ*g/ml and 100 ± 10 *μ*g/ml, respectively. Both T-24 and HT-29 cell lines were more sensitive to the effect of extract of *A. baetica* than Hep G-2 cell lines with IC_50_ 380 ± 7 *μ*g/ml. There is a significant difference between IC_50_ value induced by the methanolic extract on all treated cancerous cells (*p*^*∗∗*^ < 0.05).

In Figure 5, the methanolic extract of *A. paucinervis* roots exerts antiproliferative effects on Hep G-2 and HT-29 cell lines with IC_50_ approximately 52 ± 5 *μ*g/ml and 30 ± 6 *μ*g/ml, respectively. The T-24 cell lines were more sensitive to organic extract of *A. paucinervis* roots than Hep G-2 and HT-29 with IC_50_ 6 ± 1 *μ*g/ml. To perform the comparison, there is a significant difference between IC_50_ value induced by the methanolic extract of *A. paucinervis* roots on the treated cells (T-24, HT-29, and Hep G-2) (*p*^*∗∗*^ < 0.05).

### 3.6. Acute Toxicity Studies

The findings of acute toxicity study showed the absence of mortalities and signs of toxicity on the oral treated mice with both 2000 mg/kg of the methanolic extract of *A. baetica* roots and 2000 mg/kg methanolic extract of *A. paucinervis* roots. Slight changes in general behavior like running after the gavage compared to the control group were observed.

## 4. Discussion

Since prehistoric times, the medicinal plants have played a central role in the prevention and the treatment of various diseases [19]. In the current work, we decided to scientifically highlight the traditional uses of Moroccan *A. baetica* and *A. paucinervis* roots, such as to investigate their chemical profile, antioxidant activity, antiproliferative effect, and acute toxicity.

The phytochemical profile of the two studied plants in this work revealed the presence of alkaloids polyphenols, flavonoids, tannins, saponins, and the absence of anthraquinone, sterols, and terpenes. These results were in accordance with those reported in earlier reports which showed that the phytochemical screening of *A. longa* and *A. baetica* roots revealed the presence of phenols, flavonoids, saponins, and the absence of anthraquinones, sterols, and triterpenes [17, 20]. The qualitative test of the aerial parts of *A. indica* showed positive tests for terpenes, saponins, tannins, and flavonoids [21].

Plants with high amounts of phytoconstituents such as flavonoids and polyphenols are reported to exhibit antioxidant properties [16]. In the current research work, the total polyphenolic content was quantified using the Folin–Ciocalteau assay [22]. The rate of total polyphenolic content of methanolic extract of *A. baetica* and *A. paucinervis* roots was estimated at 360 ± 20 mg GAE/g and 280 ± 27 mg GAE/g, respectively. The total flavonoids content of *A. baetica* and *A. paucinervis* extracts were estimated at 35 ± 8 mg QE/g and 235 ± 7 mg QE/g. These findings were partly comparable to those reported in previous literature [21], in which it was reported that the total polyphenolic and flavonoids content shown in aqueous extract of *A. indica* was 25.11 ± 0.18 GAE/g and 30.41 ± 0.19 QE/g, respectively.

In the present investigation, a proposing DPPH radical scavenging activity was noticed for the methanolic extract of *A. baetica* and *A. paucinervis* with IC_50_ values of 150 ± 8 *μ*g/ml and 160 ± 10 *μ*g/ml, respectively. This property could be attributed to presence of interesting total polyphenolic content [23]. The antioxidant activity of polyphenols is due to their redox properties which could play a central role in capturing and neutralizing free radicals in order to prevent their harmful effects [24]. To the best of our knowledge, no previous data were available for *A. baetica* and *A. paucinervis* antioxidant activity. Thus, other plants of genus *Aristolochia* were phytochemically and biologically evaluated to perform comparison. The obtained results were in confirmation with early reports concerning DPPH radical scavenging activity of *A. longa* organic extract which showed a DPPH IC_50_ value of IC_50_ equal 125.40 ± 2.40 *μ*g/mL [25].

Regarding the antiproliferative activity, the results showed that the methanolic extract of *A. baetica* roots exhibited a high antiproliferative effect on T-24, HT-29, and Hep G-2 cell lines with IC_50_ approximately of 48 ± 5 *μ*g/ml, 100 ± 10 *μ*g/ml, and 380 ± 7 *μ*g/ml, respectively. On the other hand, the methanolic extract of *A. paucinervis* showed IC_50_ values of 52 ± 5 *μ*g/ml, 30 ± 6 *μ*g/ml, and 6 ± 1 *μ*g/ml on Hep G-2, HT-29, and T-24, respectively. To perform the comparison, it was reported that the antiproliferative effect of *A. baetica* organic extracts was estimated at IC_50_: 216.06 ± 15 *μ*g/mL [8]. The remarkable antiproliferative effect resulted in methanolic extracts of the studied plants was in accordance with previous data [26], in which it was reported that the IC_50_ value of *A. longa* aqueous extract against human cancer lines was determined at 15,63 *μ*g/ml.

Dysregulation of apoptosis is one of the most important factors intervened in cancer treatment [27]. The control of cancer growth still related to the ability of cancer cells to undergo apoptosis [28]. The mechanism by which genus *Aristolochia* could induce cell death was investigated in previous literature [29], it was reported that the aqueous extract of *A. longa* induces apoptosis through the mitochondrial intrinsic pathway in BL41. This mechanism could involve in our treated cell lines with methanolic extracts of the studied plants. The phytochemical screening revealed the presence of polyphenols, alkaloids, flavonoids, and tannins. On the other hand, the pharmacological activities of plants rely on their chemical compounds, and thus, the antiproliferative activity of the studied plants is associated with the detected compounds [30]. The detected flavonoids could be the responsible compounds for the antiproliferative activity [31].

Mass spectrometry plays a crucial role in the development of the organic field because of its efficiency in the qualitative and quantitative determination of organic molecules. Gas chromatography coupled to a mass spectrometer (GC-MS) permits a mixture of small molecules rather than organic constituent of low molecular weight to be analyzed [32]. Ten compounds namely pseudocumene, tetracyclo [3.3.1.0.1(3,9)] decan-10-one, *p*-vinylguaiacol, 2-epi-*trans*-*β*-caryophyllene, guaia-6,9-diene, pacifigorgiol, maaliol, methylglucose, isoaromadendrene epoxide, and *trans*-sinapyl alcohol were identified in methanolic extract of *A. baetica* using GC-MS. On the other hand, seven compounds such as dihydro-4,4,5,5-tetramethyl 2(3H)-furanone, mesitylene, dodecane, maaliol, 2-palmitoylglycerol, dioctyl terephthalate, phthalic acid, and di(2 propylpentyl) ester were detected from the methanolic extract of *A. baetica* roots.

The pharmacological and biological properties of the studied plants in the current work seem to be attributed to the identified compounds. This can be a result of the action of a single molecule or by a synergy between all these molecules without excluding the potentiation effects. As an example, pacifigorgiol found in the methanolic extract of *A. baetica* was reported in previous literature to exhibit antibacterial activities [33]. A cytotoxic effect against brine shrimp nauplii *Artemia salina* was attributed to dioctyl terephthalate detected in the methanolic extract of *A. paucinervis* [34].

The acute toxicity of the investigated methanolic in this work was useful to provide information concerning the safety control for potential uses in single administration. No clinical signs nor mortalities occurred in animals treated orally with 2000 mg/kg of the two plant extracts compared to control groups. Therefore, the predicted LD_50_ of the studied plant extracts would be higher than 2000 mg/kg. On the other hand, these extracts seem to be safe for animals with oral administration of single doses up to 2000 mg/kg under the conditions of acute toxicity. The present results were consolidated to other researches which showed the safety of single administered doses to mice (lower than 4 g/kg) [17, 35]. In the same way, the methanolic extract of *A. baetica* and *A. paucinervis* roots was not toxic with single doses according to the scale of Viala [32]. However, the genus *Aristolochia* exhibits high toxicity when ingested for a long time with repeated doses [36, 37].

## 5. Conclusion

Phytochemical profile of both *A. baetica* and *A. paucinervis* was useful to provide information on the potential of these plants as a promising source of secondary metabolites, hence, encouraging their natural potential as a source of various therapeutic agents for the treatment of cancer.

## Figures and Tables

**Table 1:** Qualitative phytochemical screening of *A. baetica* and *A. paucinervis* roots.

Phytoconstituents	*A. baetica* roots	*A. paucinervis* roots
Polyphenols	++	+++
Alkaloids	+++	++
Flavonoids	+++	++
Anthraquinone	−	−
Sterols and terpenes	−	−
Saponins	+	+
Tannins	+++	++

+++: strong positive test; ++: positive test; +: low positive test; −: negative test.

**Table 2:** Phytocomponents identified from methanolic extract of *A. baetica* roots using GC-MS.

S. No	Retention time (min)	Compound name	Molecular formula
1	6,249	Pseudocumene	C_9_H_12_
2	9,917	Tetracyclo [3.3.1.0.1(3,9)] decan-10-one	C_10_H_12_O
3	11,118	*p*-Vinylguaiacol	C_9_H_10_O_2_
4	12,902	2-epi-*trans*-*β*-Caryophyllene	C_15_H_24_
5	13,294	Guaia-6,9-diene	C_15_H_24_
6	13,76	Pacifigorgiol	C_15_H_26_O
7	14,103	Maaliol	C_15_H_26_O
8	15,11	Methylglucose	C_7_H_14_O_6_
9	15,163	Isoaromadendrene epoxide	C_15_H_24_O
10	18,466	*trans*-Sinapyl alcohol	C_1_7H_24_O_9_

**Table 3:** Phytocomponents identified from methanolic extract of *A. paucinervis* roots using GC-MS.

S. No	Retention time (min)	Compound name	Molecular formula
10	4,893	Dihydro-4,4,5,5-tetramethyl 2(3H) furanone	C_11_H_16_O_4_
17	6,246	Mesitylene	C_9_H_12_
22	9,467	Dodecane	C_12_H_26_
26	14,095	Maaliol	C15H_26_O
35	18,087	2-Palmitoylglycerol	C_19_H_38_O_4_
39	19,447	Dioctyl terephthalate	C_24_H_38_O_4_
41	19,712	Phthalic acid, di(2 propylpentyl) ester	C_24_H_38_O_4_

## Data Availability

All data are available in the following laboratories: Laboratory of Chemistry, Biochemistry, Nutrition, and Environment, Faculty of Medicine and Pharmacy, University Hassan II, Casablanca, Morocco; Research Unit and Medical Biology, National Center for Nuclear Energy, Science and Technology, CNESTEN. Rabat 10001, Morocco; Laboratory REMTEX, Higher School of Textile and Clothing Industries, Km 8, Route d'EL JADIDA, Casablanca, Morocco; and Faculty of Sciences Ben M'Sik, University Hassan II Casablanca. B. P. 7955, Morocco Laboratory of Engineering and Materials LIMAT, Faculty of Sciences Ben M'Sik, University Hassan II Casablanca. B. P. 7955, Morocco.

## References

[1] Lewis W. H. (1977). *Medicinal Botany Plants Affecting Man’s Health*.

[2] Verpoorte R. (1999). Chemodiversity and the biological role of secondary metabolites, some thoughts for selecting plant material for drug development. *Bioassay Methods in Natural Product Research and Drug Development*.

[3] Gupta M., Mazumder U. K., Kumar T. S., Gomathi P., Kumar R. S. (2004). Antioxidant and hepatoprotective effects of *Bauhinia racemosa* against paracetamol and carbon tetrachloride induced liver damage in rats. *Iranian Journal of Pharmacology and Therapeutics*.

[4] Bachiri L., Labazi N., Daoudi A. (2015). Etude ethnobotanique de quelques lavandes marocaines spontanées. *International Journal of Biological and Chemical Sciences*.

[5] Mathew S., Abraham T. E. (2006). *In vitro* antioxidant activity and scavenging effects of Cinnamomum verum leaf extract assayed by different methodologies. *Food and Chemical Toxicology*.

[6] Newman D. J., Cragg G. M. (2007). Natural products as sources of new drugs over the last 25 years⊥. *Journal of Natural Products*.

[7] Bourhia M., Shahat A. A., Almarfadi O. M. (2019). Ethnopharmacological survey of herbal remedies used for the treatment of cancer in the greater Casablanca-Morocco. *Evidence-Based Complementary and Alternative Medicine*.

[8] Chaouki W., Leger D. Y., Eljastimi J., Beneytout J.-L., Hmamouchi M. (2010). Antiproliferative effect of extracts from *Aristolochia baetica* and *Origanum compactumon* human breast cancer cell line MCF-7. *Pharmaceutical Biology*.

[9] Ouarghidi A., Martin G. J., Powell B., Esser G., Abbad A. (2013). Botanical identification of medicinal roots collected and traded in Morocco and comparison to the existing literature. *Journal of Ethnobiology and Ethnomedicine*.

[10] Lewis W. H., Elvin Lewis M. P. F. (1977). *Medical Botany. Plants Affecting Man’s Health*.

[11] Kadhim E. J. (2014). Phytochemical investigation and hepato-protective studies of Iraqi *Bryonia dioica* (Family Cucurbitaceae). *International Journal of Pharmacy and Pharmaceutical Sciences*.

[12] Belkacem N., Djaziri R., Lahfa F., El-Haci I. A., Boucherit Z. (2014). Phytochemical screening and *in vitro* antioxidant activity of various *Punica granatum* L. peel extracts from Algeria: a comparative study. *Phytothérapie*.

[13] Benariba N., Djaziri R., Bellakhdar W. (2013). Phytochemical screening and free radical scavenging activity of *Citrullus colocynthis* seeds extracts. *Asian Pacific Journal of Tropical Biomedicine*.

[14] Senhaji B., Chebli B., Mayad E. (2017). Phytochemical screening, quantitative analysis and antioxidant activity of *Asteriscus imbricatus* and *Pulicaria mauritanica* organic extracts. *International Food Research Journal*.

[15] Maisuthisakul P., Suttajit M., Pongsawatmanit R. (2007). Assessment of phenolic content and free radical-scavenging capacity of some Thai indigenous plants. *Food Chemistry*.

[16] Makrane H., El Messaoudi M., Melhaoui A., El Mzibri M., Benbacer L., Aziz M. (2018). Cytotoxicity of the aqueous extract and organic fractions from *Origanum majorana* on human breast cell line MDA-MB-231 and human colon cell line HT-29. *Advances in Pharmacological Sciences*.

[17] Bourhia M., Haj Said A. A., Chaanoun A. (2019). Phytochemical screening and toxicological study of *Aristolochia baetica* linn roots: histopathological and biochemical evidence. *Journal of Toxicology*.

[18] OECD (Organization for Economic Cooperation & Development) (2008). *OECD Guidelines for the Testing of Chemicals. Test Guidelines 425. Acute Oral Toxicity: Up-And- Down Procedure*.

[19] Assefa B., Glatzel G., Buchmann C. (2010). Ethnomedicinal uses of *Hagenia abyssinica* (Bruce) J. F. Gmel. among rural communities of Ethiopia. *Journal of Ethnobiology and Ethnomedicine*.

[20] Benarba B., Meddah B. (2014). Ethnobotanical study, antifungal activity, phytochemical screening and total phenolic content of Algerian *Aristolochia longa*. *Journal of Intercultural Ethnopharmacology*.

[21] Subramaniyan V., Saravanan R., Baskaran D., Ramalalingam S. (2015). *In vitro* free radical scavenging and anticancer potential of *Aristolochia indica* L. against MCF-7 cell line. *International Journal of Pharmacy and Pharmaceutical Sciences*.

[22] López-Vélez M., Martinez-Martínez F., Valle-Ribes C. D. (2003). The study of phenolic compounds as natural antioxidants in wine. *Critical Reviews in Food Science and Nutrition*.

[23] Shahidi F., Janitha P. K., Wanasundara P. D. (1992). Phenolic antioxidants. *Critical Reviews in Food Science and Nutrition*.

[24] Osawa T. (1994). Novel natural antioxidants for utilization in food and biological systems. *Postharvest Biochemistry of Plant Food-Materials in the Tropics*.

[25] El Omari N., Sayah K., Fettach S. (2019). Evaluation of *in vitro* antioxidant and antidiabetic activities of *Aristolochia longa* extracts. *Evidence-Based Complementary and Alternative Medicine*.

[26] Benarba B., Aoues A., Vazquez A., Ambroise G., Meddah B. (2012). *Aristolochia longa* aqueous extract triggers the mitochondrial pathway of apoptosis in BL41 Burkitt’s lymphoma cells. *International Journal of Green Pharmacy*.

[27] Chamorro M. M., Regan J. D., Opperman L. A., Kramer P. R. (2008). Effect of storage media on human periodontal ligament cell apoptosis. *Dental Traumatology*.

[28] Cohen G. M. (1997). Caspases: the executioners of apoptosis. *Biochemical Journal*.

[29] Benarba B., Meddah B., Aoues A. (2012). *Bryonia dioica* aqueous extract induces apoptosis through mitochondrial intrinsic pathway in BL41 Burkitt’s lymphoma cells. *Journal of Ethnopharmacology*.

[30] Hashemi S. R., Zulkifli I., Hair B. M. (2008). Acute Toxicity Study and Phytochemical Screening of Selected Herbal Aqueous Extract in Broiler Chickens. *International Journal of Pharmacology*.

[31] Barros L., Dueñas M., Ferreira I. C. F. R., Maria Carvalho A., Santos-Buelga C. (2011). Use of HPLC-DAD-ESI/MS to profile phenolic compounds in edible wild greens from Portugal. *Food Chemistry*.

[32] Asha K. R., Priyanga S., Hemmalakshmi S., Devaki K. (2017). GC-MS analysis of the ethanolic extract of the whole plant *Drosera indica* L.. *International Journal of Pharmacognosy and Phytochemical Research*.

[33] Izac R. R., Poet S. E., Fenical W., Van Engen D., Clardy J. (1982). The structure of pacifigorgiol, an ichthyotoxic sesquiterpenoid from the pacific gorgonian coral. *Tetrahedron Letters*.

[34] Habib M. R., Karim M. R. (2009). Antimicrobial and cytotoxic activity of di-(2-ethylhexyl) phthalate and anhydrosophoradiol-3-acetate isolated from *Calotropis gigantea* (Linn.) flower. *Mycobiology*.

[35] Bourhia M., Lahmadi A., Achtak H. (2019). Phytochemical analysis and toxicity study of *Aristolochia paucinervis* rhizomes decoction used in Moroccan alternative medicine: histopathological and biochemical profiles. *Evidence-Based Complementary and Alternative Medicine*.

[36] Cherif H. S., Saidi F., Guedioura A. (2014). Toxicological evaluation of *Aristolochia longa* L. extract in mice. *Indian Journal of Applied Research*.

[37] Benzakour G., Amrani M., Oudghiri M. (2012). A histopathological analyses of *in vivo* anti-tumor effect of an aqueous extract of *Aristolochia longa* used in cancer treatment in traditional medicine in Morocco. *International Journal of Plant Research*.

